# Difference in the joint space of the medial knee compartment between full extension and Rosenberg weight-bearing radiographs

**DOI:** 10.1007/s00330-021-08253-6

**Published:** 2021-09-07

**Authors:** Yugo Miura, Nobutake Ozeki, Hisako Katano, Hayato Aoki, Noriya Okanouchi, Makoto Tomita, Jun Masumoto, Hideyuki Koga, Ichiro Sekiya

**Affiliations:** 1 Center for Stem Cell and Regenerative Medicine, Tokyo Medical and Dental University (TMDU), 1-5-45 Yushima, Bunkyo-ku, Tokyo, Japan; 2 Kanagawa Institute of Industrial Science and Technology, 3-2-1 Sakado, Takatsu-Ku, Kawasaki, Kanagawa Japan; 3 School of Data Science, Graduate School of Data Science, Yokohama City University, 22-2, Seto, Kanazawa-ku, Yokohama, Kanagawa, Japan; 4 Fujifilm Corporation, 7-3, Akasaka 9-chome, Minato-ku, Tokyo, Japan; 5 Department of Joint Surgery and Sports Medicine, Graduate School of Medical and Dental Sciences, Tokyo Medical and Dental University (TMDU), 1-5-45 Yushima, Bunkyo-ku, Tokyo, Japan; 6 Department of Applied Regenerative Medicine, Tokyo Medical and Dental University (TMDU), 1-5-45 Yushima, Bunkyo-ku, Tokyo, Japan

**Keywords:** Knee joint, Meniscus, Cartilage, X-rays, Magnetic resonance imaging

## Abstract

**Objectives:**

Radiographs are the most widespread imaging tool for diagnosing osteoarthritis (OA) of the knee. Our purpose was to determine which of the two factors, medial meniscus extrusion (MME) or cartilage thickness, had a greater effect on the difference in the minimum joint space width (mJSW) at the medial compartment between the extension anteroposterior view (extension view) and the 45° flexion posteroanterior view (Rosenberg view).

**Methods:**

The subjects were 546 participants (more than 50 females and 50 males in their 30 s, 40 s, 50 s, 60 s, and 70 s) in the Kanagawa Knee Study. The mJSW at the medial compartment was measured from both the extension and the Rosenberg views, and the “mJSW difference” was defined as the mJSW in the Rosenberg view subtracted from the mJSW in the extension view. The cartilage region was automatically extracted from MRI data and constructed in three dimensions. The medial region of the femorotibial joint cartilage was divided into 18 subregions, and the cartilage thickness in each subregion was determined. The MME was also measured from MRI data.

**Results:**

The mJSW difference and cartilage thickness were significantly correlated at 4 subregions, with 0.248 as the highest absolute value of the correlation coefficient. The mJSW difference and MME were also significantly correlated, with a significantly higher correlation coefficient (0.547) than for the mJSW difference and cartilage thickness.

**Conclusions:**

The MME had a greater effect than cartilage thickness on the difference between the mJSW at the medial compartment in the extension view and in the Rosenberg view.

**Key Points:**

*• The difference in the width at the medial compartment of the knee between the extension and the flexion radiographic views was more affected by medial meniscus extrusion than by cartilage thickness*.

## Introduction

Osteoarthritis (OA) of the knee is associated primarily with degenerative changes in the articular cartilage [1]. Knee OA is typically diagnosed based on weight-bearing radiographs, which are the most widespread imaging tool for diagnosis and for detecting narrowing of the joint space width. The standing 45° flexion posteroanterior view (Rosenberg view) has a greater sensitivity than the standing extension anteroposterior view (extension view) for detecting OA involving the joint space width, according to a systematic review [2].

**Fig. 1:**
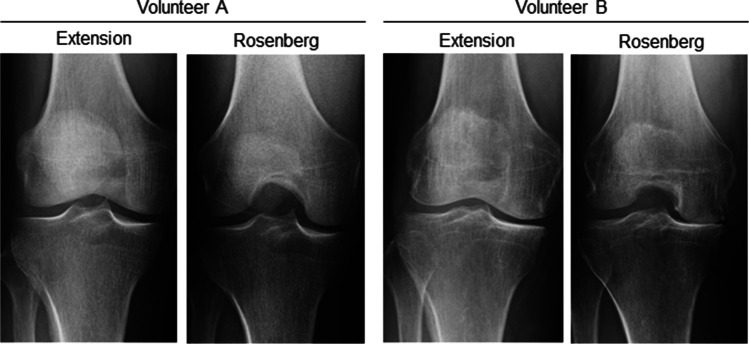
Knee radiographs of full extension and Rosenberg weight-bearing views. In volunteer A, the minimum joint space width (mJSW) at the medial compartment is similar between the full extension and the Rosenberg views. In volunteer B, the mJSW is narrower in the Rosenberg view than in the full extension view

Knee OA frequently arises due to meniscus degeneration and meniscal extrusion, which induce dysfunction in the load distribution—one of the most important functions of the meniscus [3, 4]. Medial meniscus extrusion (MME) occurs after medial meniscus injury, but it also progresses with age, even in the absence of any obvious history of trauma [5, 6]. An increasing number of reports have now shown an association between MME and a narrowing of the minimum joint space width (mJSW) at the medial compartment [4, 6–8]. The radiographic view of the mJSW at the medial compartment can appear similar in both the extension and the Rosenberg views in some subjects, but the Rosenberg view appears narrower in others (Fig. 1). Whether this difference is due to the cartilage or to the meniscus remains unclear.

Evaluation of articular cartilage typically involves arthroscopic assessment as the gold standard [9], but this is an invasive procedure and has relatively large inter- and intra-rater variabilities [10]. We have addressed this problem by developing a novel system that can automatically extract cartilage data from knee magnetic resonance imaging (MRI) data, construct it in three dimensions, and generate a cartilage thickness value with high inter- and intra-rater reliabilities [11].

The purpose of the present study was to use cross-sectional study data and MRI analysis to determine which of the two factors—cartilage thickness or MME—has a greater effect on the difference between the mJSW at the medial compartment in the extension view and in the Rosenberg view. We hypothesized that the MME had a greater effect than cartilage thickness on the difference between the mJSW at the medial compartment in the extension view and in the Rosenberg view.

## Patients and methods

### Kanagawa Knee Study and subject enrollment

The Kanagawa Knee Study had a cohort consisting of at least 50 females and 50 males in their 30 s, 40 s, 50 s, 60 s, and 70 s. Most subjects were desk workers who worked for or had retired from the Kanagawa Prefecture Government (Kanagawa is a prefecture located next to Tokyo in Japan). The aim of the study was to understand the epidemiology and the natural course of knee OA. Subjects with a history of knee OA, lower extremity trauma, previous surgery, rheumatoid arthritis, or consecutive visits to the hospital for knee disorders for more than 3 months were excluded. Knee radiographs and MRI scans were performed between September 1, 2018, and August 30, 2019. A total of 561 participants (276 females and 285 males) participated in the Kanagawa Knee Study. Six subjects with lateral knee OA were excluded, and nine subjects with inappropriate radiographs were excluded from the remaining 555 subjects, leaving a total of 546 subjects (267 females and 279 males) who were included in the current analysis. This study was approved by our institutional review board, and all patients provided written informed consent.

### Measurements of mJSW in radiographs

**Fig. 2:**
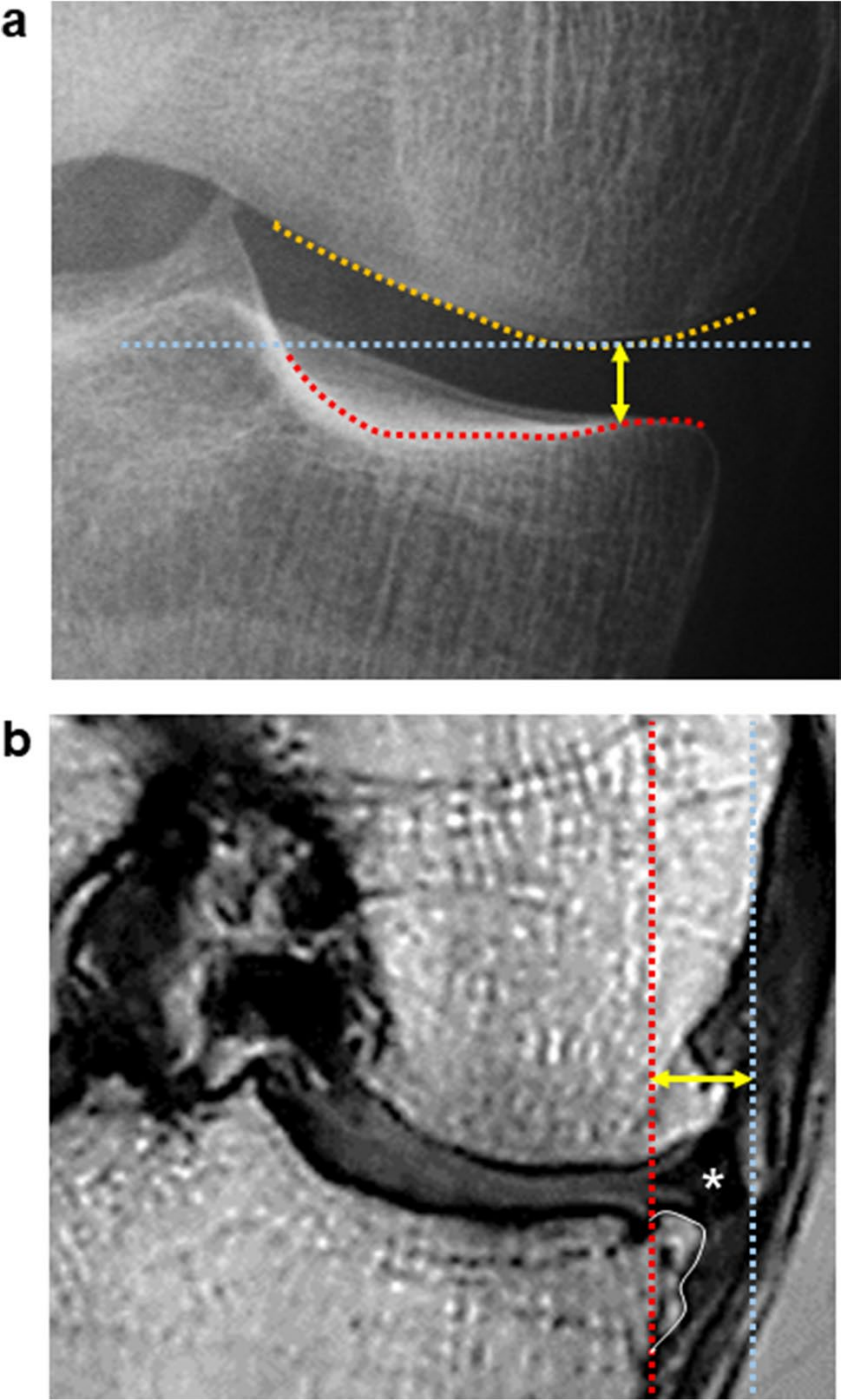
Measurement methods. **a** Measurement of the minimum joint space width by radiography. A horizontal straight line (blue line) was drawn at the lowest end of the medial femoral condyle (orange line). A bright radio-dense zone (red line), defined at the anterior edge of the medial tibia, was also drawn. The minimum distance (yellow arrow) between these two lines was defined as the minimum joint space width (mJSW). **b** Measurement of MME by MRI. Using a coronal plane MRI, the MME (yellow arrow) was defined as the distance between the perpendicular line (red line) from the medial margin of the tibia, excluding the osteophytes (orange line), and the perpendicular line (blue line) from the outer edge of the MM (star)

Radiographs of the knee were obtained using the extension view and the Rosenberg view. Digital Imaging and Communications in Medicine (DICOM) data were imported into a dedicated viewer (RadiAnt DICOM viewer 2020.1.1, Medixant) and enlarged to fit a 24-in. display. For mJSW at the medial compartment, the perceived narrowest point of the medial knee joint space was measured manually in both the extension and the Rosenberg views [6] (Fig. 2a). The difference obtained by subtracting the mJSW in the Rosenberg view from that in the extension view was then calculated and defined as the “mJSW difference.”

#### MRI

The MRI system (Achieva 3.0TX, Philips) was used at 3.0 T with 16-channel coils. The sagittal plane of the knee joint was acquired to obtain both a fat-suppressed spoiled gradient echo sequence image and a proton weighted image, with total scan durations of 7 min 30 s and 7 min 10 s, respectively. For both images, sagittal images were obtained at an in-plane resolution of 0.31 × 0.31 mm, a partition thickness of 0.36 mm (320 slices), and a field of view (head to tail × anterior to posterior) of 150 × 150 mm.

### MRI measurements of MME

The MRI DICOM data were analyzed using software (Vincent, Fujifilm). Coronal cross-section images were reconstructed from the MRI data of proton-enhanced sagittal cross-section images. The slice in which the tibia was at its maximum transverse diameter was selected. A perpendicular line was drawn from the inner edge of the tibia, excluding the osteophytes. A perpendicular line was also drawn from the outer edge of the medial meniscus (MM). The distance between these two perpendicular lines was defined as the MME [12] (Fig. 2b). The correlation between the MME and the mJSW difference in the radiographic analysis was examined.

### Measurements of cartilage thickness

**Fig. 3:**
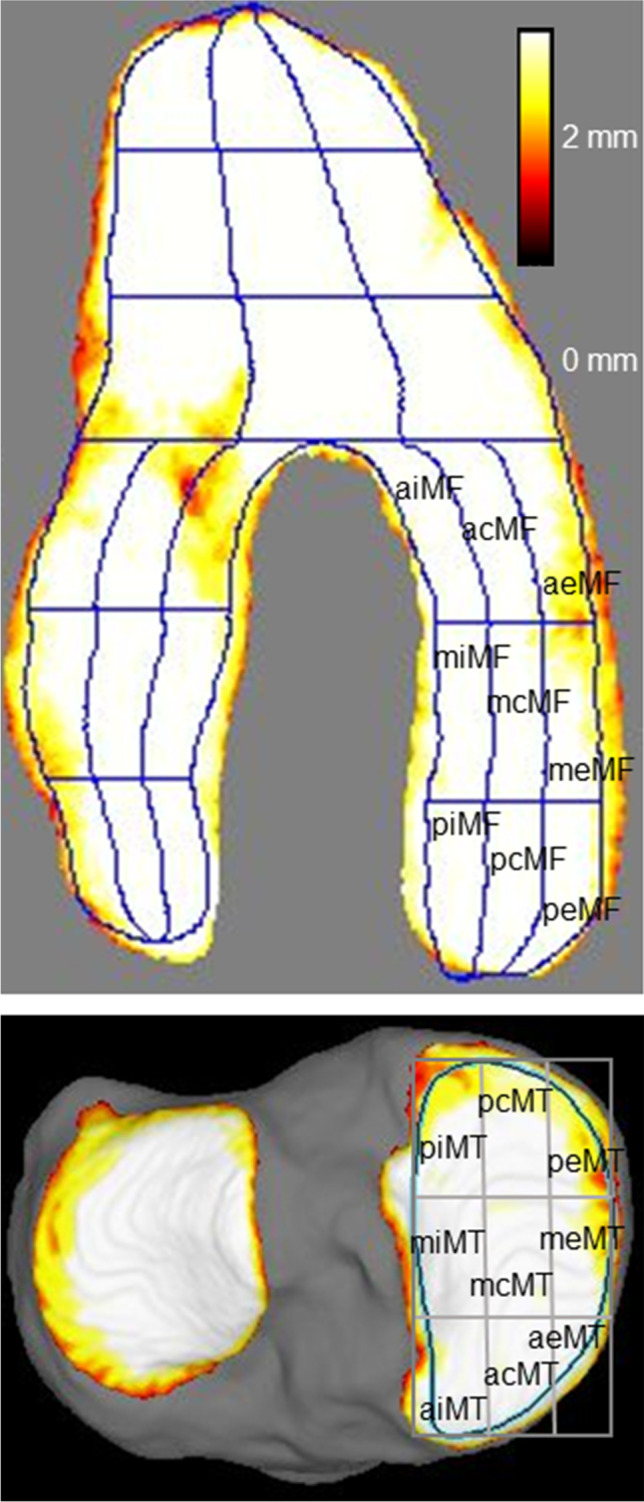
MRI cartilage thickness mapping and subregions of the knee. MF, medial femoral; aiMF, anterior internal MF; acMF, anterior central MF; aeMF, anterior external MF; miMF, middle internal MF; mcMF, middle central MF; meMF, middle external MF; piMF, posterior internal MF; pcMF, posterior central MF; and peMF, posterior external MF. MT, medial tibial; piMT, posterior internal MT; pcMT, posterior central MT; peMT, posterior external MT; miMT, middle internal MT; mcMT, middle central MT; meMT, middle external MT; aiMT, anterior internal MT; acMT, anterior central MT; and aeMT, anterior external MT

The MRI DICOM data were read by the software (Vincent, Fujifilm), the bone and cartilage regions were automatically extracted, and the tibial cartilage was projected vertically onto a plane using the long axis of the bones. The femoral cartilage was projected radially around the intercondylar axis, which connected the centers of the medial and lateral condyles. The center of the condyle was determined by approximating the condyle to an ellipse in a lateral view. The software provided cartilage thickness mapping by displaying cartilage thickness as a color scale; thick areas of cartilage were indicated in white and thin areas in red (Fig. 3). The software automatically drew a closed curve line for the ROI based on the bone contour. It then divided the ROI into 3 regions, based on the shape of the ROI, and then further divided each region into 9 subregions based on the shape of each region. In this study, only the medial compartment was analyzed.

In the medial tibial region, the software also automatically drew closed curve lines for the region of interest (ROI), based on the bone contour. The ROI was divided into nine subregions with three equal vertical and horizontal divisions (Fig. 3).

The nomenclature of each region and subregion was based on a previous report by Eckstein et al. [13]. The average cartilage thicknesses in the 18 subregions were obtained, and the correlation between the cartilage thickness and the mJSW difference at the medial compartment in the radiographic analysis was examined for each subregion.

The Dice similarity coefficient for segmentation accuracy was 0.91 for the femoral cartilage, 0.89 for the tibial cartilage, 0.91 for the ROI of the femoral subchondral bone, and 0.89 for the ROI of the medial/lateral tibia plateau [14]. Interscan measurement error of knee cartilage thickness in the 18 subregions of the medial femoral region and the medial tibial region ranged from 0.03 to 0.11 mm, and was less than 0.10 mm at 15 subregions [15].

### Statistical analysis

Student’s *t* test was used to assess the relationship between the mJSW at the medial compartment in the extension view and Rosenberg view. Pearson’s correlation coefficient was used to evaluate the association between age and mJSW, between cartilage thickness and mJSW, and between MME and mJSW. Correlation coefficients of 0.00–0.19 were considered “very weak,” 0.20–0.39 as “weak,” 0.40–0.59 as “moderate,” 0.60–0.79 as “strong,” and 0.80–1.0 as “very strong” [16].

The levels of significance for multiple Pearson’s correlation coefficients were adjusted by Bonferroni’s correction method. For age, in a set of 3 correlation coefficients, the *p* value < 0.017 (= 0.05/3) was considered statistically significant. For cartilage thickness and MME, in a set of 19 correlation coefficients (cartilage thickness at 18 subregions and MME), a *p* value < 2.63 × 10^−3^ (= 0.05/19) was considered statistically significant.

Inter-rater reliability was assessed with the intraclass correlation coefficient (ICC), and the limits of agreements (LOA) within 95% confidence interval (CI) were evaluated using Bland–Altman plots. For the mJSW in the extension view at the medial compartment, a total of 50 subjects (five females and five males in their 30 s, 40 s, 50 s, 60 s, and 70 s) were randomly selected and measured independently by two examiners using blinded radiographic data. For the MME, 526 subjects were selected and measured independently by two examiners. Statistical analyses were performed with the Statistical Package for the Social Sciences version 22 (SPSS, Inc.).

## Results

### Characteristics of subjects

The 546 volunteers consisted of 267 (49%) females and 279 (51%) males with an average age of 55 ± 14 years (average ± SD) and an average BMI of 23 ± 3 kg/m^2^. The Kellgren-Lawrence (KL) grading scale in the extension view identified 518 (94.9%) subjects with grade 0 or 1 knee OA; 12 (2.2%) with grade 2; 7 (1.3%) with grade 3; and 9 (1.6%) with grade 4.

### Inter-rater reliability of mJSW and MME

**Fig. 4:**
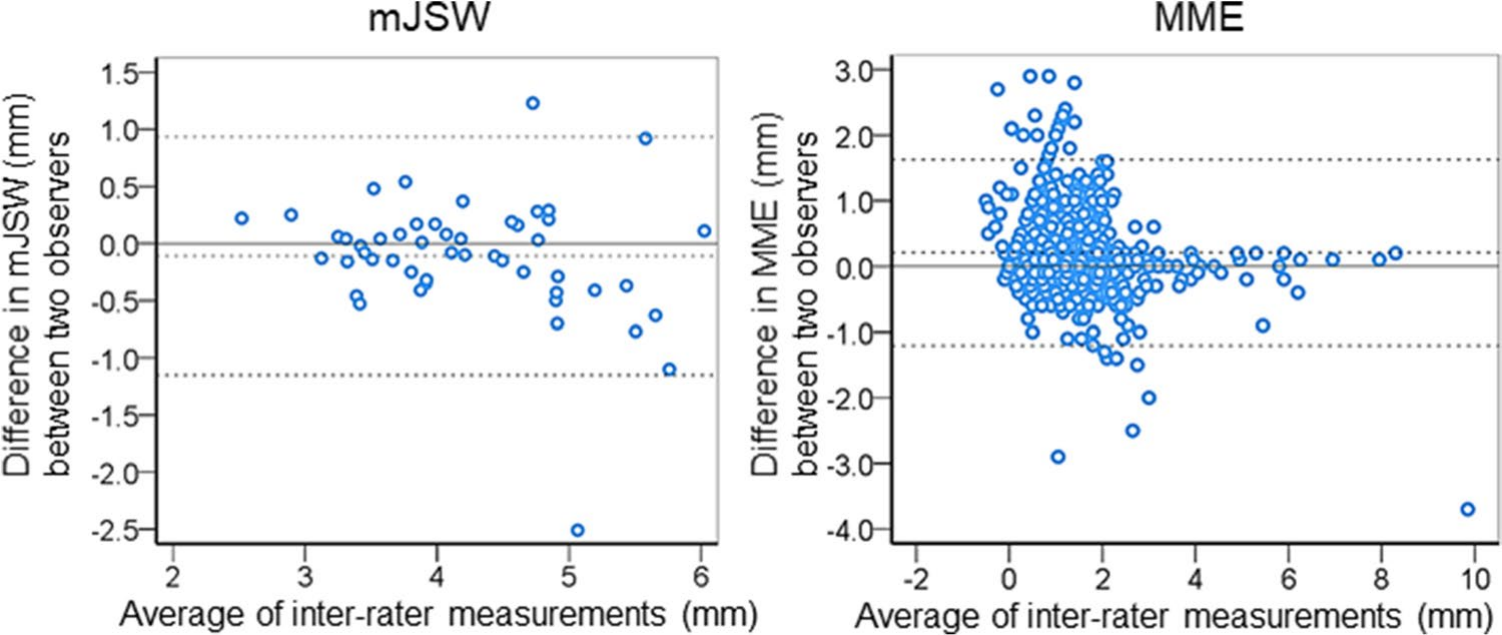
Bland–Altman plots of inter-rater agreement for mJSW and MME. The difference between the two observers is plotted against the average value of the two raters’ measurements. The middle dotted line indicates the average difference. The upper and lower dotted lines correspond to the upper and lower limits of agreement with 95% CI

The inter-rater reliability (ICC 2, 1) of the mJSW was 0.89 (95% CI, 0.81–0.94), which was considered good reliability [17]. The inter-rater reliability (ICC 2, 1) of the MME was 0.90 (95% CI, 0.87–0.92), which was considered excellent reliability [17]. For the inter-rater agreements, the average difference in mJSW was 0.11 mm, the range of the 95% LOA was − 1.2 to 0.9 mm (average ± 1.96 SD), and the number of outliers was 2 out of 50 in the Bland–Altman plot (Fig. 4). The average difference in MME was 0.2 mm, the range of 95% LOA was − 1.2 to 1.6 mm, and the number of outliers was 25 out of 526.

### The mJSW in the extension and Rosenberg views

The mJSW at the medial compartment was 4.3 ± 1.0 mm (average ± SD) in the extension view and 4.2 ± 1.0 mm in the Rosenberg view (*n* = 546), and the difference of 0.1 ± 0.9 mm between them was statistically significant (*p* = 0.03).

### Correlation between age and mJSW

**Fig. 5:**
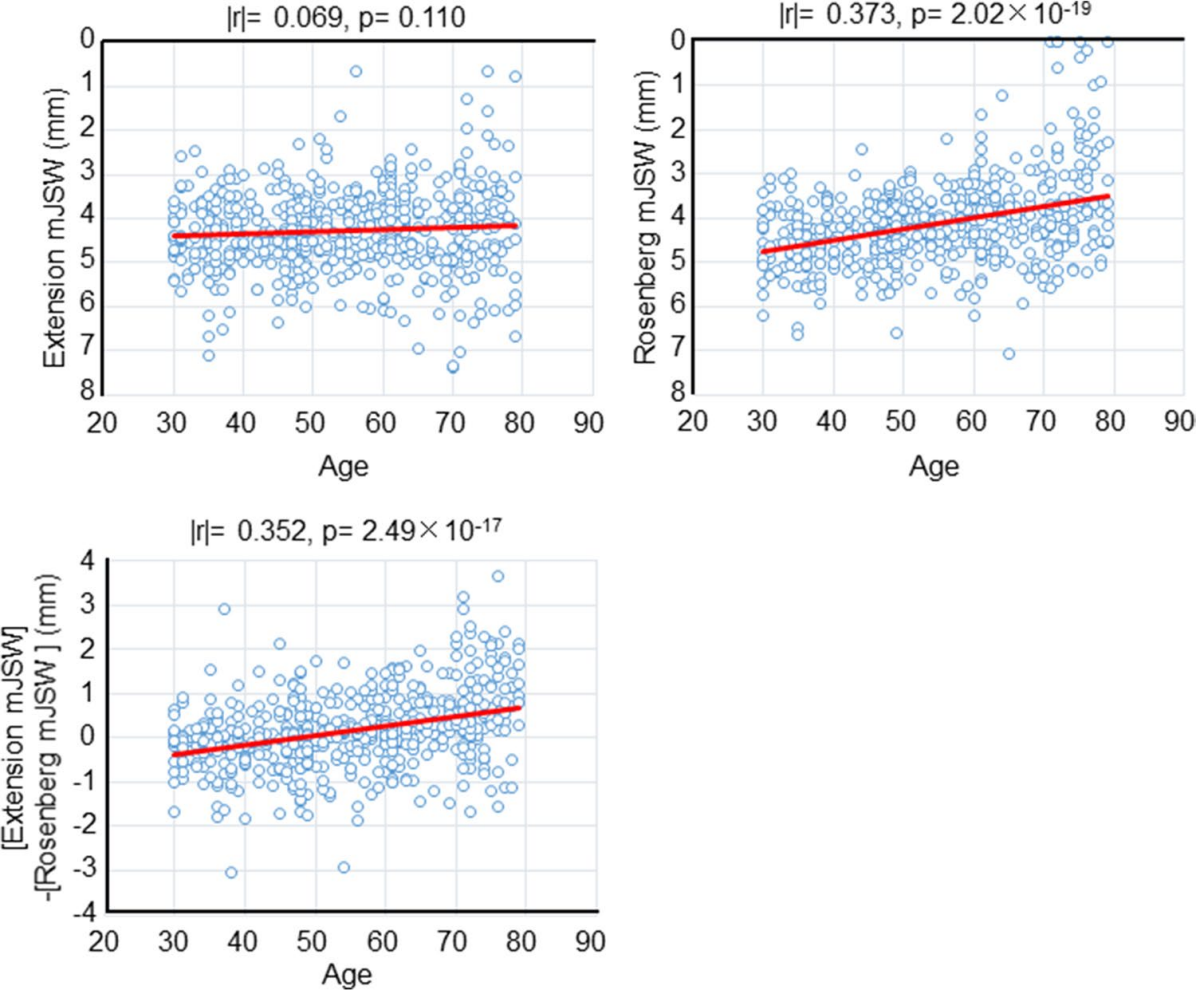
Correlation between the mJSW and age. Scatterplots show age and mJSW at the medial compartment in the extension view, between age and mJSW in the Rosenberg view, and between age and “the difference obtained by subtracting the mJSW in the Rosenberg view from mJSW in the extension view.” Absolute values of the correlation coefficient (|*r*|) and *p* values are also shown

The mJSW in the extension view was not correlated with age (Fig. 5), whereas the mJSW in the Rosenberg view and the mJSW difference showed weak but significant correlation with age [16] (Fig. 5). No significant difference was found between the two correlation coefficients that were correlated with age.

### Correlation between “cartilage thickness” and mJSW

**Fig. 6:**
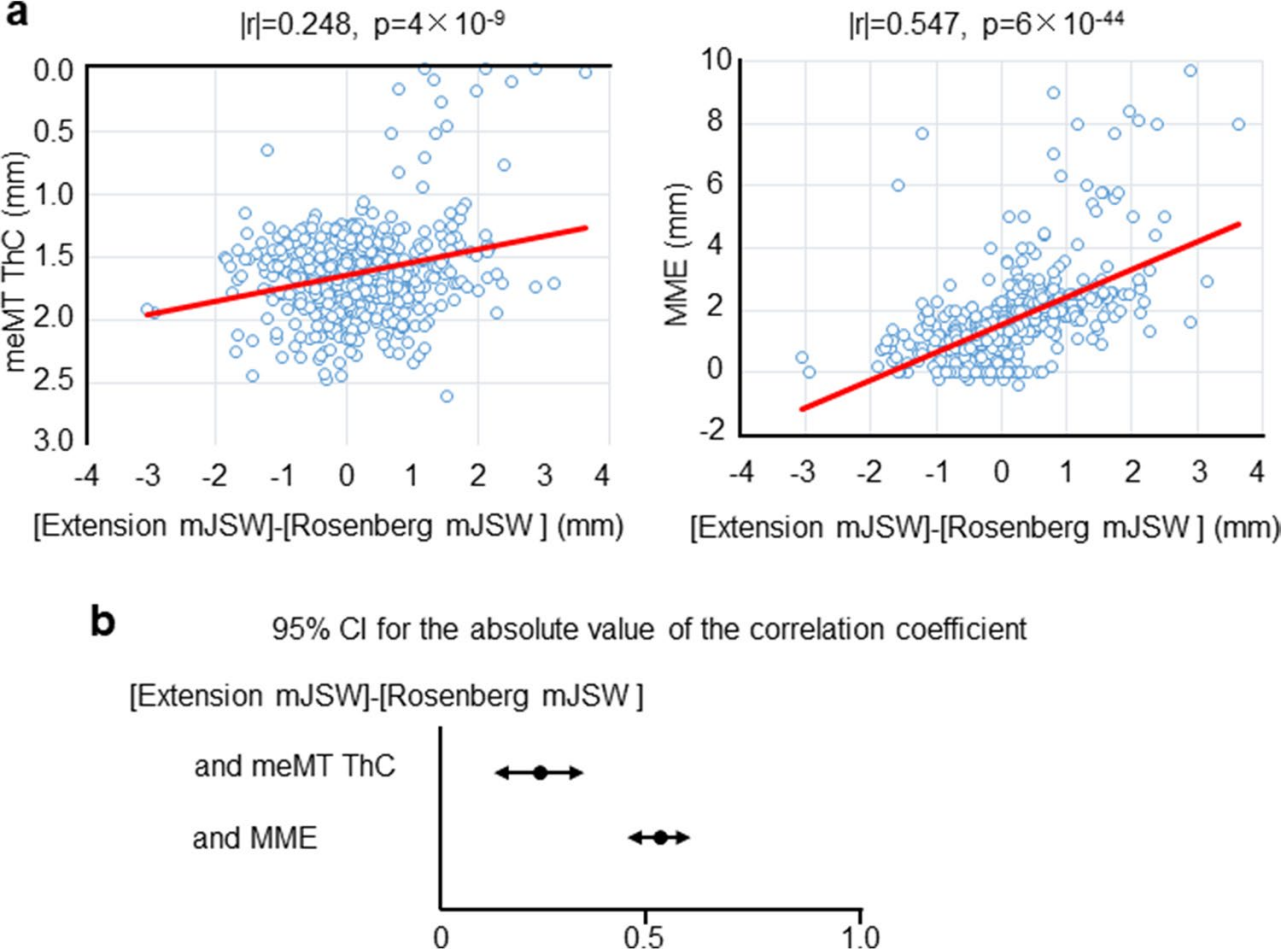
Correlation between the mJSW difference and cartilage thickness and between the mJSW difference and MME. **a** Scatterplots between “the difference obtained by subtracting the mJSW at the medial compartment in the Rosenberg view from mJSW in the extension view” and “cartilage thickness (ThC) at meMT subregion” and between “the difference obtained by subtracting the mJSW in the Rosenberg view from the mJSW in the extension view” and the medial meniscus extrusion (MME). Absolute values of the correlation coefficient (|*r*|) and *p* values are shown. **b** 95% CI for the absolute value of the correlation coefficient

The mJSW difference and the cartilage thickness were significantly correlated at miMF and mcMF (very weak correlation) in the medial femoral cartilage [16] (Table 1). They were also significantly correlated at meMT and mcMT (weak correlation) in the medial tibial cartilage [16]. The greatest correlation coefficient was found for the meMT with a value of 0.248 (95% CI, 0.168–0.326) (Fig. 6a).

**Table 1:** Correlations between “[extension mJSW] − [Rosenberg mJSW] (mm)” and “cartilage thickness (mm)” at different subregions in the medial femoral and tibial cartilage (*n* = 546)

Subregion				Subregion			
Medial femoral	Correlation coefficient		*p* value	Medial tibial	Correlation coefficient		*p* value
peMF	0.008		0.852	peMT	0.120		0.005
meMF	0.009		0.829	meMT	0.248	*	4 × 10^−9^
aeMF	0.004		0.930	aeMT	0.078		0.068
pcMF	0.091		0.033	pcMT	0.090		0.036
mcMF	0.142	*	9 × 10^−4^	mcMT	0.234	*	3 × 10^−8^
acMF	0.059		0.168	acMT	0.000		0.991
piMF	0.013		0.770	piMT	0.010		0.813
miMF	0.147	*	6 × 10^−4^	miMT	0.039		0.369
aiMF	0.013		0.770	aiMT	0.094		0.028

Each correlation coefficient is expressed as an absolute value

Abbreviations: *MF* medial femoral condyle, *aiMF* anterior internal MF, *acMF* anterior central MF, *aeMF* anterior external MF, *miMF* middle internal MF, *mcMF* middle central MF, *meMF* middle external MF, *piMF* posterior internal MF, *pcMF* posterior central MF, *peMF* posterior external MF, *MT* medial tibial, *piMT* posterior internal MT, *pcMT* posterior central MT, *peMT* posterior external MT, *miMT* middle internal MT, *mcMT* middle central MT, *meMT* middle external MT, *aiMT* anterior internal MT, *acMT* anterior central MT, *aeMT* anterior external MT

^*^p < 2.63 × 10^−3^

### Correlation between MME and mJSW

The overall MME was 1.7 ± 1.4 mm (*n* = 546). The mJSW difference and MME were significantly correlated, with an absolute value of 0.547 for the correlation coefficient, indicating a moderate correlation (95% CI, 0.486–0.603) [16] (Fig. 6a). This correlation coefficient was significantly greater than the coefficient value between the mJSW difference and the cartilage thickness at the meMT subregion, where the greatest correlation coefficient was found among the 18 subregions in the medial femoral and tibial cartilages.

## Discussion

The most important finding of the present study was that the effect on the mJSW difference was greater for the MME than for the cartilage thickness. We examined the cartilage thickness and the MME to determine which of the two had a greater effect on the mJSW difference at the medial compartment. The highest absolute value of the correlation coefficient between the mJSW difference and cartilage thickness at meMT was 0.248. The absolute value of the correlation coefficient between the mJSW difference and the MME was 0.547, which was significantly greater than the maximum correlation coefficient (0.248) associated with cartilage thickness. We hypothesized that the MME had a greater effect than cartilage thickness on the mJSW difference at the medial compartment, and our hypothesis was correct using cross-sectional study data and MRI analysis.

We divided the medial compartment into 18 subregions for the evaluation of cartilage thickness, and we examined the correlation coefficient between the mJSW difference at the medial compartment and the cartilage thickness at each subregion. The absolute value of this correlation coefficient exceeded 0.2 only for the meMT and mcMT, which were located in the center of the medial tibial cartilage in the anterior–posterior direction. Fluoroscopic analysis by Feng et al. previously showed that the contact point of the medial femoral joint moved from a central to a posterior location on the femoral side as it flexed from extension to flexion, whereas the tibial side remained central [18]. This suggests that the cartilage loss associated with OA occurs from the center of the tibial articular surface, in support of our results.

The radiographs were taken in the standing position, but the MRIs were taken in the usual supine position in our study. If the MRIs had been taken in the standing position, the results could be different. For example, Marsh et al. reported that the difference in medial femorotibial cartilage thickness between standing and supine MRI examinations was 0.2 mm for KL0 and 0.4 mm for KL2 [19]. Conversely, Kawaguchi et al. measured MME by ultrasonography and reported a difference between the standing and supine positions of 0.4 mm in KL0 and 0.5 mm in KL2 [20]. These results suggest that the effects of the standing position are greater for the MME than for the cartilage thickness. Therefore, we would have expected to see a stronger correlation with the mJSW difference in the MME than in the cartilage thickness had the MRIs been performed in the standing position in our study.

The medial meniscus of the normal knee moves posteriorly when the knee is flexed. Since the posterior horn is less mobile than the anterior horn, the MME on flexion is smaller in the posterior segment than in the anterior segment [21, 22]. Once injury or degeneration of the body or the root of the MM occurs, the MME increases [3]. Our present study demonstrated the relationship between the mJSW difference observed in radiographs and the MME view in MRI. Further clarification of the meniscus damage and degeneration using MRI will more clearly establish the significance of the mJSW difference.

The clinical implication of our findings is that the increase in MME can be detected by comparing the mJSW between the extension view and Rosenberg view. At present, the onset of knee OA is considered to reflect an increase in MME (possibly caused by degeneration of the MM or MM posterior root tears) and a subsequent decrease in the coverage of the MM on the articular cartilage surface [20]. MRI can directly evaluate the MME, but since it is not commonly performed in the standing position, it may underestimate the MME. Ultrasound can directly evaluate the MME in the standing position, but it is not usually the first choice for knee imaging. Therefore, if the MME could be detected using only a plain radiographic examination, which is a routine diagnostic imaging procedure for the knee, this would be very useful in clinical practice.

Our study had three limitations. One was that the study population was not randomly selected from the general population and was therefore subject to selection bias. The Kanagawa Knee Study recruited volunteers from desk workers and excluded those with a history of lower extremity trauma, surgery, or disease with more than 3 months of hospital visits. The study included 546 knees ranging in age from 30 to 79 years, with only 2.9% having KL3–4. Unlike previous studies, the mJSW of the medial compartment in the extension view did not correlate with age in this study [5]. The reason is probably that this population had a milder degree of knee OA than is found in the general population.

Another limitation is that the mJSW in the radiographs and MME in MRI were measured using a manual procedure. Although each of the inter-rater reliabilities was acceptable, the measurement error cannot be ignored. By contrast, the cartilage thickness was automatically analyzed using the MRI analysis software.

A third limitation is that analysis of the sum of the femoral and tibial cartilage thicknesses, rather than the cartilage thickness at only one tibia subregion (meMT), might have been more appropriate. However, choosing the best tibial subregion that contacts a single femoral subregion from nine medial tibial subregions is not easy using anatomical knowledge.

In conclusion, the difference in the joint space of the medial knee compartment between the conventional standing extension anteroposterior view and the Rosenberg view (i.e., the mJSW difference) was more affected by medial meniscus extrusion than by cartilage thickness.

## Abbreviations

CI: Confidence interval
DICOM: Digital Imaging and Communications in Medicine
ICC: Intraclass correlation coefficient
KL: Kellgren-Lawrence
LOA: Limits of agreement
mJSW: Minimum joint space width
MM: Medial meniscus
MME: Medial meniscus extrusion
MRI: Magnetic resonance imaging
OA: Osteoarthritis
ROI: Region of interest
SD: Standard deviation

## Declarations

### Guarantor:

The scientific guarantor of this publication is Tetsushi Furukawa.

### Statistics and biometry:

One of the coauthors, Makoto Tomita PhD (School of Data Science, Graduate School of Data Science, Yokohama City University, Kanagawa, Japan), is a specialist in statistics.

### Methodology:

• retrospective • prognostic study • performed at one institution
